# Case report: Low-grade myofibroblastic sarcoma resembling lymphoma on ^18^F-FDG PET/CT

**DOI:** 10.3389/fonc.2023.1139720

**Published:** 2023-03-07

**Authors:** Huan Zhang, Liu He, Bin Hu, Xiaoming Zhang, Lichun Zheng

**Affiliations:** ^1^ Department of Nuclear Medicine, Tangshan Gongren Hospital, Tangshan, Hebei Province, China; ^2^ Graduate School of North China University of Science and Technology, Tangshan, Hebei, China; ^3^ Graduate School of Hebei Medical University, Shijiazhuang, Hebei, China

**Keywords:** myofibroblastic sarcoma, cancer, FDG, PET/CT, metastasis

## Abstract

Low-grade myofibroblastic sarcoma is a rare malignant tumor that arises from mesenchymal tissue and affects the head (particularly the tongue and oral cavity) and neck. It is characterized by local recurrence, with metastases being uncommon. We present a 72-year-old man who initially complained of a painless and progressive mass in the right inguinal region and came for consultation, and a malignant tumor was suspected based on the clinical and pelvic MRI manifestations. The ^18^F-FDG PET/CT revealed that the multiple lesions were located in the mediastinum, retroperitoneum, pelvis, and inguinal lymph nodes; hence, lymphoma was considered to be a combination of the symptoms. However, the histology of the ultrasound-guided puncture indicated low-grade myofibroblastic sarcoma. The patient was next administered chemotherapy, but the lesions did not undergo remission.

## Introduction

It is common knowledge that positron emission tomography with computed tomography (PET/CT) utilizing ^18^F-fluoro-deoxyglucose (^18^F-FDG) is a fairly effective examination for identifying and evaluating malignant tumors. Low-grade myofibroblastic sarcoma (LGMS) has a very low incidence. Such little literature exists on LGMS and ^18^F-FDG PET/CT imaging of LGMS. In our case, a LGMS patient with ^18^F-FDG PET/CT abnormalities including lymph nodes in multiple areas of the body had distant metastases. The ^18^F-FDG PET/CT imaging presentation was mistaken for lymphoma, and the imaging distinction between lymphoma and LGMS is addressed further in the discussion. In addition, the tumor did not diminish appreciably despite receiving two chemotherapy treatments, confirming the LGMS is low susceptibility to chemotherapy.

## Case description

A 72-year-old man came to our hospital for evaluation with a painless mass in the right inguinal region which had been growing for three months. The mass lacked softness and immobility and was rigid and smooth. The patient has no previous history of tumor or surgery, and no family history of related diseases. The tumor makers revealed a slightly elevated neuron-specific enolase level of 73.09 ng/ml (reference range: 0-29.13 ng/ml) and ferritin level of 582.4ng/ml (reference range: 30-400 ng/ml), in addition to the serum levels of lactic dehydrogenase and creatine kinase, which were 551 U/L (reference range: 114-240 U/L). Other than the indicators listed above, all other laboratory test indicators were within normal range. The pelvic MRI (pictures not shown) revealed multiple enlarged lymph nodes surrounding the right pelvis, right inguinal area, and right common iliac arteries; a malignant tumor was suspected in combination with clinical and imaging characteristics.

**Figure 1:**
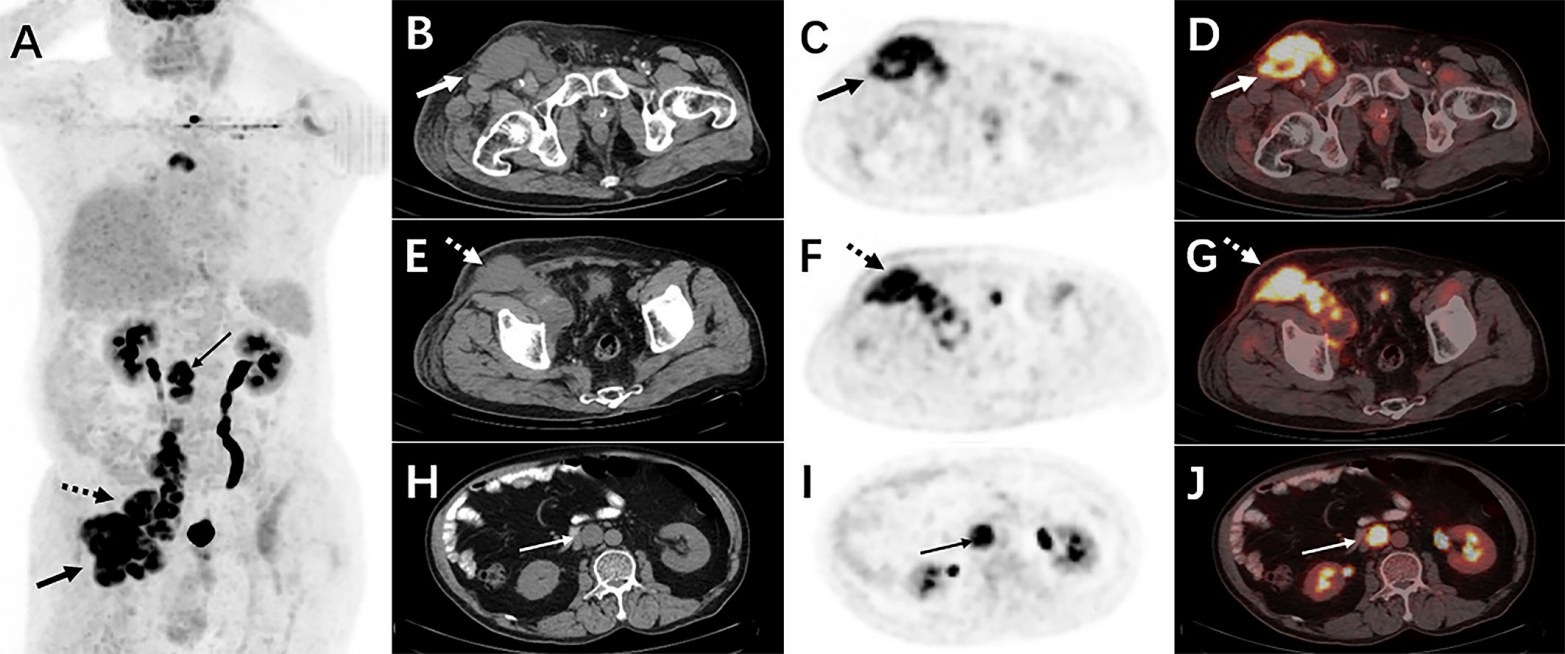
The ^18^F-FDG PET/CT imaging before treatment. The MIP picture **(A)** revealed hypermetabolic foci in the retroperitoneum and mediastinum (arrows). On the axial images, several low-density lesions were detected in the right inguinal region (SUVmax 12.1)(solid arrows) (**B**: CT, **C**: PET, **D**: fusion). The image (**E**: CT, **F**: PET, **G**: fusion) revealed numerous lymph nodes with abnormal metabolic confluence and a SUVmax of 9.8. (dotted arrows). Some retroperitoneal hypermetabolic foci (SUVmax 10.2) were found (thin arrows) (**H**: CT, **I**: PET, **J**: fusion).

**Figure 2:**
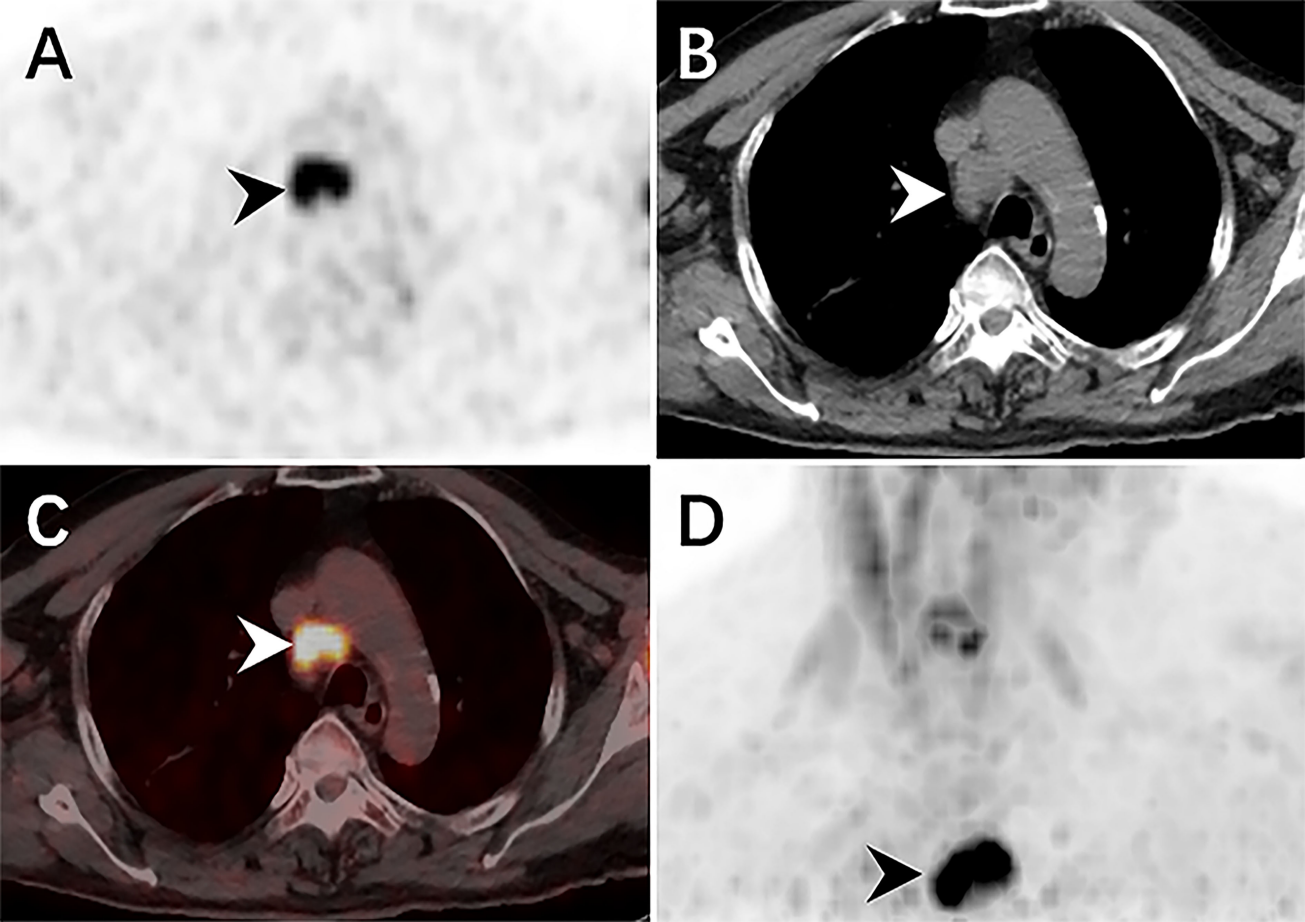
The delay ^18^F-FDG PET/CT revealed elevated activity foci in the mediastinum, with a SUVmax of 7.0. The CT scan revealed a single, low-density lymph node nearby the aortic arch (arrowheads) (**A**: PET, **B**: CT, **C**: fusion, **D**: MIP).

Therefore, an ^18^F-FDG PET/CT was conducted to evaluate the general status of the patient. Except for multiple significantly enhanced ^18^F-FDG activity foci in the pelvic cavity and inguinal area, the maximum intensity projection (MIP) image (**Figure 1A**) revealed abnormal activity in the mediastinum and retroperitoneum (arrows). On the axial images (**Figure 1B**: CT, **C**: PET, **D**: fusion), several hypermetabolic foci were observed in the right inguinal region, which corresponded to heterogeneous low-density enlarged lymph nodes (solid arrows), and the maximum standardized uptake value (SUVmax) of the lesion was 12.1. Moreover, the maximum cross-sectional area of the huge bulk was 9.4 cm by 6.0 cm. The abdomen axial imaging (**Figure 1E**: CT, **F**: PET, **G**: fusion) revealed multiple lymph nodes of activity (dotted arrows) along the right common iliac vessels and parts of lesions accompanied by fusion. In the axial pictures, a lymph node with elevated activity (thin arrows) surrounding the descending aorta (SUVmax, 10.2) (**Figure 1H**: CT, **I**: PET, **J**: fusion). A delay ^18^F-FDG PET/CT imaging was conducted since the moving affected the picture quality, and one of the mediastinum foci disappeared due to the moving’s influence. Nevertheless, the axial imaging demonstrated an elevated ^18^F-FDG lymph node activity around the aortic arch (arrows), with a SUVmax of 7.1. (**Figure 2A**: PET, **B**: CT, **C**: fusion, **D**: MIP). All of the above ^18^F-FDG PET/CT imaging indicated lymphoma.

**Figure 3:**
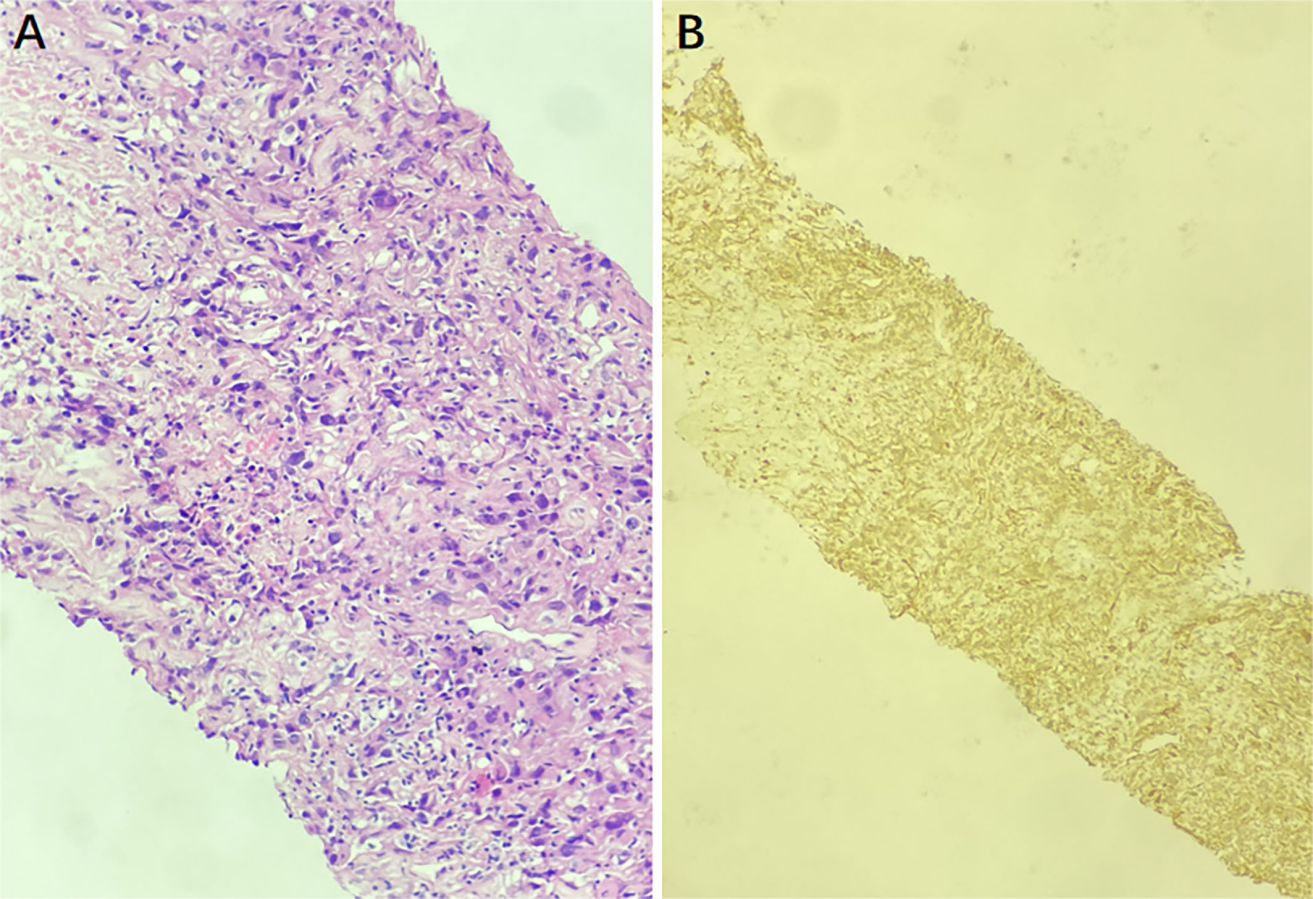
The puncture biopsy specimen pathological findings show infiltration of myofibroblasts, and there is no inflammatory cells (hematoxylin-eosin ×200) **(A)**. The immunohistochemistry staining for SMA was positive, indicating a LGMS **(B)**.

Then ultrasound-guided puncture biopsy of the right inguinal mass was performed, and the pathology was low-grade myofibroblastic sarcoma(LGMS). Histopathological examination (hematoxylin-eosin 200) of the specimens obtained from the right inguinal mass revealed spindle-shaped cells arranged in fascicles (**Figure 3**). Positive immunohistochemistry stains were observed for SMA, TTF-1, and Vimentin, while negative staining was observed for CK, CK20, CK7, MyoD1, Myogenin, P63, and S100. All of the findings were consistent with LGMS.

**Figure 4:**
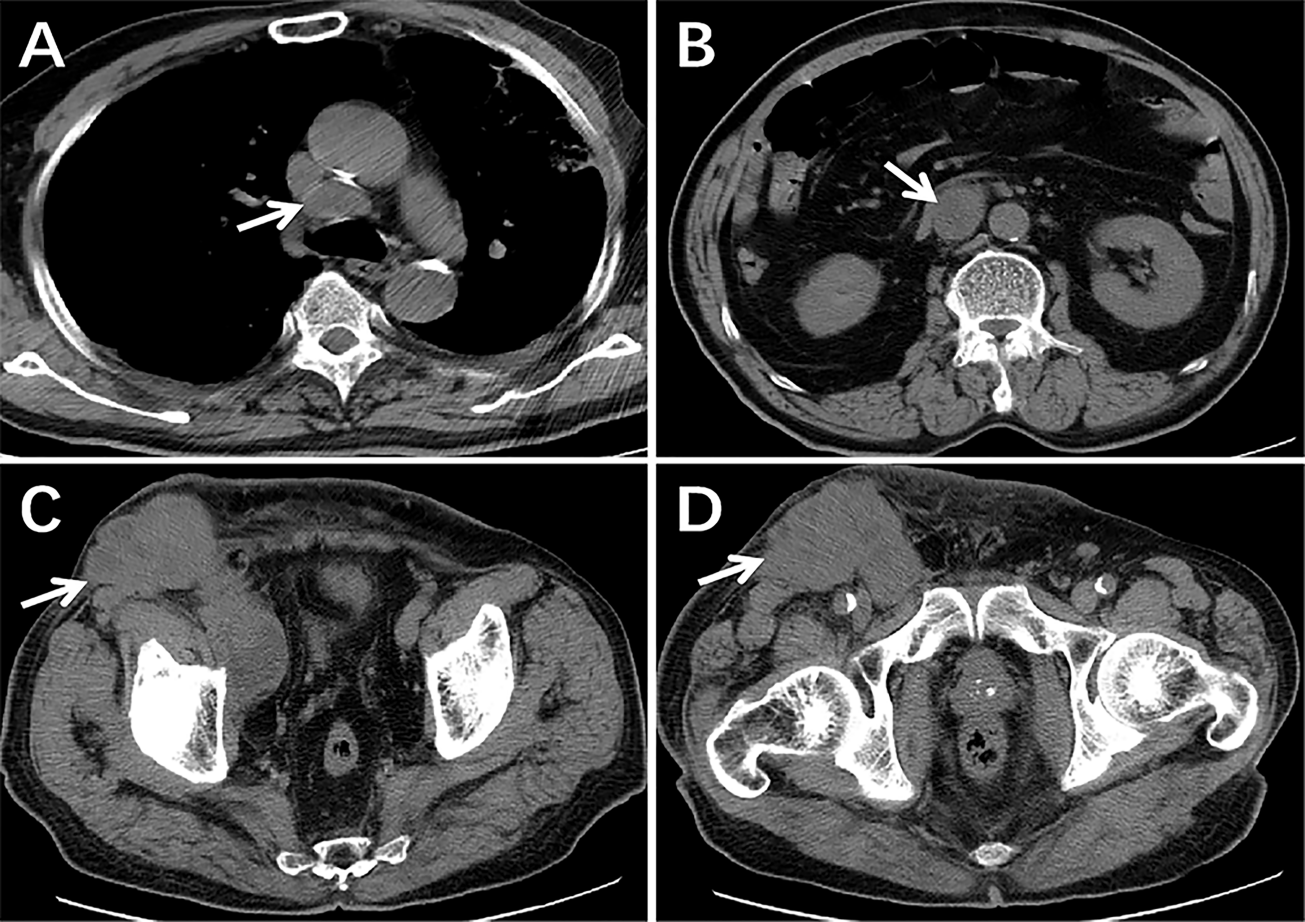
The CT scans following two cycles of treatment. There were still lesions in the mediastinum, retroperitoneum, pelvic, and inguinal regions and the size and quantity of lymph nodes had not decreased significantly from pre-treatment imaging (arrows) **(A–D)**.

Furthermore, the patient successfully completed two cycles of ifosfamide and bevacizumab chemotherapy. The CT of the chest, abdomen and pelvic cavity indicated no change in the quantity or volume of lesions, and the largest mass measured approximately 8.9cm × 7.1cm (**Figure 4**). Due to the lack of a significant reduction in lesions following treatment, the patient abandoned treatment and died the next year. Informed consent has been obtained for this retrospective study.

## Discussion

LGMS is a rare and low-grade malignant bone and soft tissue tumor that originates from mesenchymal tissue. The most common patients are older males, and the clinical presentation is a painless, progressive enlargement resembling lymphoma symptoms. The definitive diagnosis of LGMS is determined by histopathology. LGMS has a varied immunophenotype, but typically displays myogenic antibodies similar to SMA. In addition, Vimentin is the specific marker for fibrosarcoma, and it is positive in the majority of LGMS patients. The immunohistochemistry staining of SMA and Vimentin was positive in our case.

LGMS mainly involves the head and neck, particularly the oral cavity and tongue (1–4). LGMS can be observed in the bone, muscle, breast, and pleura in addition to a few individual case reports (5–10). However, very few studies have reported lymph node involvement in LGMS. And it is generally accepted that LGMS has a higher risk of recurrence and a lower metastatic incidence. But our research revealed that a patient with LGMS had lymph nodes in the abdominal cavity, pelvic cavity, and mediastinum region. According to a study (11), ^18^F-FDG PET/CT is of considerable value in the diagnosis of LGMS and effectively detects occult soft tissue malignancy (12). On the ^18^F-FDG PET/CT images, our case demonstrates intense ^18^F-FDG activity foci corresponding to multiple enlarged lymph nodes with mutual fusion throughout the body, similar to lymphoma. Through further review of the literature (13), we found the most notable distinctions between this case of LGMS and typical lymphoma: lymphoma involving lymph nodes is generally homogeneous in density and mostly symmetrical in distribution, whereas LGMS had apparent liquefied necrosis and was located on one side in this case. Additionally, the lymph nodes involved in lymphoma are frequently distributed bilaterally in the inguinal region, whereas in this case, the lymph nodes involved in the LGMS were located on one side only. Lymphoma involving large lymph nodes is typically created by the fusion of numerous lymph nodes, but in this case, there is no sign of fusion in the right inguinal mass, supporting the notion that this is the primary focus. Therefore, LGMS should be suspected if numerous lymph nodes with necrosis especially were involved on ^18^F-FDG PET/CT.

Other diseases, in addition to lymphoma, should be considered in the differential diagnosis. Firstly, lymph node hypertrophy is a result of inflammation. In this case, the patient had no fever symptoms and no abnormal laboratory testing for inflammatory indices, therefore inflammation was ruled out. The second differential diagnosis is myxofibrosarcoma. The main difference between the two from a pathological perspective is that myxofibrosarcoma had more mucus, whereas the pathology specimen from our patient had no mucus. Additionally, SMA, a distinctive myogenic antibody that aids in differentiating between the two, is typically present in LGMS.

The research findings demonstrate that complete surgical resection of the lesion is the most effective treatment for LGMS and generally has a promising prognosis (14), but LGMS is not sensitive to radiotherapy and chemotherapy (9). In our study, however, the patient was elder and had extensive lesions, so his body could not tolerate surgery and he chose chemotherapy. Furthermore, there was no substantial reduction of the lesion after two cycles of ifosfamide and bevacizumab chemotherapy, confirming that chemotherapy is insensitive to LGMS and has poor therapeutic efficacy.

In our case, lymphoma was suspected based on either the clinical symptoms or the ^18^F-FDG PET/CT imaging. Nevertheless, the patient was identified with LGMS after undergoing a biopsy. Even though LGMS has a very low incidence, the disease is potentially severe. Therefore, early diagnosis and therapy are essential for the prognosis of LGMS patients, and we should consider the possibility of LGMS when a patient presents with multiple lymph node involvement.

## Data availability statement

The original contributions presented in the study are included in the article/supplementary material. Further inquiries can be directed to the corresponding author.

## Ethics statement

The studies involving human participants were reviewed and approved by the Institutional Tangshan Gongren Hospital. The patients/participants provided their written informed consent to participate in this study.

Written informed consent was obtained from the individual(s) for the publication of any potentially identifiable images or data included in this article.

## Author contributions

HZ wrote the manuscript. LH, BH and XZ preformed image acquisition and prepared clinical data. LZ reviewed the manuscript. All authors contributed to the article and approved the submitted version.
